# The bioaccessibility of phenolic compounds, nutritional quality, and textural properties of gluten‐free muffins enriched with artichoke leaves and green lentil protein isolate

**DOI:** 10.1111/1750-3841.17626

**Published:** 2025-01-19

**Authors:** Zehra Gulsunoglu‐Konuskan, Sevgi Deren Yagdi, Burcu Ersoy

**Affiliations:** ^1^ Faculty of Health Sciences, Nutrition and Dietetics Department Istanbul Aydin University Istanbul Turkey

**Keywords:** artichoke leaf, gluten‐free muffin, in vitro digestion, green lentil protein

## Abstract

**Abstract:**

Although the gluten‐free market is expanding and offers a variety of products, there are still some deficiencies in the nutritional and sensory quality of these products. Therefore, this study explores the bioaccessibility of phenolic compounds, nutritional quality, and textural properties of gluten‐free muffins enriched with artichoke leaves and green lentil protein (GLP) isolate, two novel ingredients introduced together for the first time in this context. The incorporation of GLP isolate aims to enhance the protein content, while artichoke leaves are evaluated for its potential to improve phenolic content and antioxidant activity. The muffins were also subjected to in vitro digestion to assess the bioaccessibility of phenolic compounds. The results demonstrated that the addition of GLP isolate increased the protein content 1.4 times, while artichoke leaves contributed to enhance the total phenolic content (TPC), flavonoid content (TFC), antioxidant activity (TAA), and ash content by 2.1, 5.4, 3.2–3.5, and 1.3 times, respectively. Nevertheless, the addition of artichoke leaves increased the hardness, gumminess, and chewiness of gluten‐free muffins (*p *< 0.05). The bioaccessibility values of gluten‐free muffins varied between 138% and 220%, 142% and 206%, and 40% and 160% for TAA, TPC, and TFC, respectively. Chlorogenic acid, ferulic acid, and quercetin derivatives were found as main phenolic compounds in gluten‐free muffins enriched with artichoke leaves in undigested and all phase of digestion. This study provides valuable insights into the development of functional gluten‐free muffins, highlighting the potential of artichoke leaves and GLP isolate as innovative ingredients in gluten‐free food products.

**Practical Application:**

This study shows how adding green lentil protein and artichoke leaves to gluten‐free muffins can make them healthier by boosting their protein, antioxidants, and other nutrients. However, while muffins became more nutritious, their texture changes. Food companies could use this information to improve gluten‐free products, but they may need to adjust the recipe to improve the sensory properties.

## 1 INTRODUCTION

Celiac disease, affecting approximately 1% of the global population, is an immune‐mediated disorder triggered by the ingestion of gluten, a protein found in wheat, rye, and barley. This immune response damages the small intestine's lining, resulting in nutrient malabsorption and various symptoms, including diarrhea, abdominal pain, weight loss, fatigue, anemia, and bloating (Dhillon et al., 2023). The primary treatment involves adhering to a strict gluten‐free diet, leading to increased interest in gluten‐free product development. Additionally, some non‐celiac individuals also adopt gluten‐free diets, perceiving them as healthier options (Jeong et al., 2021).

Despite the availability of various gluten‐free formulations, many commercial gluten‐free products are of low quality. These products, often starch‐based, tend to stale quickly and lack certain nutrients, such as fiber, protein, and minerals (Çelik & Isik, 2023; Dhillon et al., 2023). Gluten, which imparts elasticity, cohesiveness, and viscosity to dough, is challenging to replace, causing gluten‐free products to suffer from poor texture, reduced volume, and rapid staling. Efforts to improve these products focus on using conventional and novel ingredients to substitute gluten and employing technological approaches, such as flour pretreatments, sourdough fermentation, use of hydrocolloids and starches, addition of plant‐based proteins and enzyme, to enhance aeration, flavor, and aroma (Houben et al., 2012; Jeong et al., 2021).

Bakery foods like bread, biscuits, and muffins are globally popular due to their varied taste, long shelf life, and low cost. They also serve as excellent carrier for fortifying with macro‐ and micronutrients (Dhillon et al., 2023). Especially, muffins that are easy to make gluten‐free muffins with similar characteristics to wheat‐containing muffins by substituting wheat flour with gluten‐free flours, though they often result in lower protein content, but higher in fat and carbohydrate contents. Thus, both the sensory and nutritional properties of gluten‐free muffins need improvement (Jeong et al., 2021). For this reason, legumes are mainly used as a gluten‐free alternative due to their high protein, dietary fiber, and mineral contents. They provide a well‐balanced amino acid profile and can enhance the nutritional value of gluten‐free products. Incorporating legumes into baked products can also improve texture and mouthfeel (Jeong et al., 2021). Studies have explored the use of cowpea (Shevkani et al., 2015), chickpea (Shaabani et al., 2018; Tomić et al., 2022), and soy and pea protein isolates (Matos et al., 2014) to enhance the functional and nutritional quality of gluten‐free bakery products.

Rice (*Oryza sativa*) flour has emerged as a popular alternative for gluten‐free baking. One of the primary advantages of rice flour is its neutral flavor, which allows it to blend seamlessly into various recipes without overpowering other ingredients. Additionally, rice flour has a fine texture that can contribute to a desirable crumb structure in baked goods, helping to achieve a light and airy muffin. Nutritionally, rice flour is a source of carbohydrates; however, it is relatively low in dietary fiber compared to whole grain flours. To enhance the fiber content of gluten‐free muffins, rice flour can be combined with other fiber‐rich ingredients, such as pearl millet (Dhillon et al., 2023), corn flour (Sciammaro et al., 2018), inulin, guar gam, and oat fiber (Gularte, de la Hera, et al., 2012). These combinations not only improve the nutritional profile of the muffins but also aids in moisture retention and texture improvement, resulting in a more satisfying product. In this study, quinoa flour was used to produce gluten‐free muffins with the combination of rice flour. Quinoa (*Chenopodium quinoa* Willd.), a pseudo‐cereal, is also highly nutritious, containing significant amounts of protein (14%–16%), fiber, vitamins, and minerals (Lorusso et al., 2017). It can safely be used in gluten‐free diets and offers health benefits such as regulating cholesterol and glucose levels (Gulsunoglu‐Konuskan et al., 2021).

Artichoke (*Cynara cardunculus* L. var. *scolymus*) is widely cultivated in the Mediterranean and the top producer is Egypt (459,962 t/year) followed by Italy (378,110 t/year) and Spain (200,070 t/year). Turkey takes the ninth place with the production amount of 40,815 t/year (FAOSTAT, 2022). The capitulum, or head, is the edible part of the plant and is mainly consumed fresh, frozen, or canned. The stems and outer leaves, which account for about 80%–85% of the plant's total fresh weight, are the primary by‐products generated during industrial processing of artichoke (Colantuono et al., 2018). Artichoke by‐products are recognized as excellent sources of dietary fiber and polyphenols (Pandino et al., 2013), which can improve the antioxidant activity and functional properties of bakery products, as suggested by some researchers (Boubaker, Damergi, et al., 2016; Cannas et al., 2024; Colantuono et al., 2018; Díaz et al., 2019; Vacca et al., 2023). However, more research is needed to validate the bioefficacy of these bioactive compounds in food products. Food processing and structure significantly affect the chemical composition and bioavailability of bioactive compounds in enriched‐food products (Dziki et al., 2014). The bioaccessibility, bioavailability, and potential antioxidant effects of polyphenols from artichoke heads have been previously studied both in vivo and in vitro (D'Antuono et al., 2015; Domínguez‐Fernández et al., 2021). However, there is still a lack of scientific literature on the bioaccessibility and potential bioefficacy of bioactive compounds in foods enriched with artichoke by‐products.

The aim of this study was to determine the effect of different addition levels (5%, 10%, and 15%) of artichoke leaves and 10% green lentil protein (GLP) isolate on the chemical composition and physical properties of rice and quinoa‐based gluten‐free muffins. Additionally, the study evaluates the potential bioaccessibility of polyphenols and the overall antioxidant capacity of the muffins during in vitro gastrointestinal digestion.

## 2 MATERIALS AND METHODS

### 2.1 Materials

Artichoke outer leaves were obtained from a local outer market in Istanbul, Turkey. The chemical composition of artichoke leaves was analyzed and the results were found as 1.6 ± 0.0% lipid, 13.9 ± 0.7% protein, 81.1 ± 0.9% carbohydrate, and 3.4 ± 0.1% ash. Rice (1.4% lipid, 75.2% carbohydrate, 7.2% protein, and 0.1% salt), white quinoa (5.0% lipid, 24.5% carbohydrate, and 17.1% protein) and green lentil (1.5% lipid, 53.3% carbohydrate, 21.7% protein, and 0.6% salt) were purchased from local grocery store in Istanbul, Turkey, and the chemical compositions of them taken from the label of commercial products. The other ingredients for the muffins such as eggs, milk (regular milk with %3 fat), sunflower oil, corn starch, sugar, and baking powder were sourced from local suppliers in Istanbul, Turkey. Chemicals used for proximate analysis were of analytical grade and chemicals for HPLC analysis were of high purity. All chemicals obtained from Sigma‐Aldrich.

### 2.2 Preparation of ingredients

The rice and quinoa flour for gluten‐free muffins was prepared by milling using a stainless‐steel grinder (IKA) and sifting through a sieve of 130–200 µm opening. Artichoke outer leaves were dried using a freeze drier (Teknosem) for 24 h at 0.001 mBar (−55°C). The dried artichoke leaves were finely ground to a particle size of <200 µm using a grinder and then stored at −20°C for muffin production.

### 2.3 Preparation of GLP isolates

GLP isolate was obtained by isoelectric precipitation method described by Lee et al. (2021) with some modifications. Green lentil seeds were first ground with a stainless‐steel grinder and sieved through a 200 µm mesh size to obtain fine green lentil flours. To obtain GLP, 100 g of green lentil flour was dispersed in 1 L of distilled water and stirred with a magnetic stirrer (IKA) at 150 rpm for 16 h at 18°C. After that, the pH of solution was adjusted to 9.0 ± 0.05 with 1 N NaOH before mixing with magnetic stirrer at 150 rpm for 1 h at room temperature. The slurry was centrifuged at 4700 rpm for 10 min at 10°C to collect the supernatant. Then, the pH of supernatant was adjusted to 4.5 ± 0.05 with 1 N HCl for protein precipitation, and the solution was stirred for another 30 min using a magnetic stirrer. The precipitated protein was collected after centrifugation at 4700 rpm for 10 min at 10°C and washed twice with deionized water. The precipitated protein was freeze‐dried at 0.001 mBar (−55°C) for 24 h to obtain GLP powders. The protein content of GLP isolate was determined using Kjeldahl method and calculated as 89.3 ± 0.9% in dry matter (dm). The GLP isolate was stored at −20°C till further analysis.

### 2.4 Preparation of gluten‐free muffins

Gluten‐free muffins were prepared according to the method developed by Shevkani and Singh (2014). The ingredients and the amounts used in the production of gluten‐free muffins are given in Table 1. Solid ingredients (quinoa flour, rice flour, GLP isolate, artichoke leaves, baking powder, and corn starch) were added in the same bowl and then mixed with liquid ingredients. Egg yolk was mixed with sugar and whisked with a mixer (Sinbo). Egg white was whisked until it turned to a white cloud and mixed gently with egg yolk and other liquid ingredients (sunflower oil and milk). Solid ingredients were included to the liquid part and mixed with a mixer. Finally, a semi‐liquid muffin batter was obtained, and approximately 30 g of batter was weighed to circular mold (diameter = 50 mm) and then baked 20 min at 150°C in an electrical oven (Esty). Muffins were let for cooling and stored in a plastic bag during 24 h at ambient temperature before analysis.

**TABLE 1:** Formulation of the gluten‐free muffins enriched with green lentil protein (GLP) and artichoke leaves.

Ingredients (g)	Control (C)	Control for GLP (PC)	Artichoke leaves		
			5% (AL5)	10% (AL10)	15% (AL15)
Rice flour	16	14.4	13.6	12.8	12
Quinoa flour	16	14.4	13.6	12.8	12
GLP isolate	0	3.2	3.2	3.2	3.2
Artichoke leaves	0	0	1.6	3.2	4.8
Whole egg	16	16	16	16	16
Milk (regular milk with 3% fat)	16	16	16	16	16
Sunflower oil	16	16	16	16	16
Baking powder	1	1	1	1	1
Sugar	16	16	16	16	16
Corn starch	3	3	3	3	3

In this study, total five formulations were designed in terms of protein isolate and artichoke leaves compositions. Quinoa and rice flour was used as control, C (50% quinoa flour + 50% rice flour). Note that 10% of GLP isolate was replaced with the same amount of quinoa and rice flour, which is called as the protein control, PC (45% quinoa flour + 45% rice flour + 10% GLP). The remaining three formulations, labeled as AL5, AL10, and AL15, were prepared with varying proportions of quinoa flour, rice flour, and artichoke leaves. Specifically:

- AL5 contains 42.5% quinoa flour + 42.5% rice flour + 10% GLP + 5% artichoke leaves.
- AL10 contains 40% quinoa flour + 40% rice flour + 10% GLP + 10% artichoke leaves.
- AL15 contains 37.5% quinoa flour + 37.5% rice flour + 10% GLP + 15% artichoke leaves.

In each of these formulations, the amount of quinoa and rice flour was adjusted accordingly to accommodate the different amounts of artichoke leaves.

### 2.5 Physical properties of gluten‐free muffins

The weight loss of gluten‐free muffins was calculated by subtracting weight of muffin before baking and 2‐h‐cooled muffin after baking, and the height was measured with a digital caliper from the highest point of the muffin (Matos et al., 2014). The volume, symmetry, and uniformity index of gluten‐free muffins were determined using a template given in AACC (10–91) method (AACC, 2000).

The color of gluten‐free muffins was measured in crust and crumb parts using a Hunter colorimeter (CR‐400 Minolta) measuring L*, a*, and b* color parameters. L* presents values from black (L* = 0) to white (L* = 100); values of a* parameter varies from red (+ values) to green (− values) and b* parameters vary from yellow (+ values) to blue (– values). The color differences between samples and control (ΔE*) were also determined.

Texture parameters of gluten‐free muffins were carried out according to Matos et al. (2014). Briefly, the analysis was conducted on gluten‐free muffin (approximately 30 mm in length and 50 mm in diameter) using a TA Plus Texture analyzer (Lloyd) equipped with a 25 mm diameter stainless‐steel cylindrical probe and a 50 N load cell. A double compression test (texture profile analysis) was conducted at a probe compression rate of 1 mm/s, applying 50% sample deformation during both compressions. Hardness, chewiness, gumminess, cohesiveness, and springiness values of gluten‐free muffins were determined. The analyses were conducted three times.

### 2.6 Proximal composition of gluten‐free muffins

The moisture content of the gluten‐free muffins was determined by gravimetric methods based on AOAC (2005). To avoid any degradation of phenolic compounds, the muffins were dried with a freeze dryer for 24 h at 0.001 mBar (−55°C). The freeze‐dried gluten‐free muffins were then finely ground using a stainless‐steel grinder, and the ground muffins were used for ash, protein and lipid analysis. The ash content was measured using a gravimetric method as described in AOAC (2005). Lipid content was determined using Soxhlet method and the dried gluten‐free muffins extracted during 5 h with petroleum ether (Boubaker, Damergi, et al., 2016). Protein content was quantified using the Kjeldahl method (AOAC, 2005) and a conversion factor of 6.25 was applied to calculate crude protein content from nitrogen. To calculate the total carbohydrate content of the gluten‐free muffins, the weights of lipids, proteins, and ash were deducted from the total weight of the dried muffins (Tomić et al., 2022).

### 2.7 Extraction of phenolic compounds from undigested gluten‐free muffins

Phenolic compounds were extracted using an 80% methanol aqueous solution with solvent‐to‐sample ratio of 10:1. Phenolic compounds were extracted from 1 g of the samples with 10 mL of methanol solution according to Petropoulos et al. (2018). The solid and solvent mixture was placed in an ultrasonic bath (Protech) for 30 min at ambient temperature. Afterward, the mixture was centrifuged at 4000 rpm for 10 min at 4°C, and the supernatant was collected. The supernatants were then filtered using a Whatman No. 4 filter paper and stored at −18°C for further analysis.

### 2.8 In vitro gastrointestinal digestion model

In vitro gastrointestinal digestion of gluten‐free muffins was performed according to Minekus et al. (2014). This method was carried out in three stages as oral, gastric, and intestinal digestion to evaluate the bioaccessibility of phenolic compounds found in gluten‐free muffins enriched with artichoke leaves. Saliva, gastric, and intestinal fluids contained ammonium carbonate, hydrochloric acid, magnesium chloride hexahydrate, monopotassium phosphate, potassium chloride, sodium bicarbonate, and sodium chloride, and all digestive fluids were prepared based on the protocol. Before starting, all the simulated digestive fluids were heated to 37°C in an oven. To simulate the oral digestion, 2 g of lyophilized gluten‐free muffins was mixed with 3.5 mL saliva fluid, 0.5 mL amylase (1500 U/mL), and 25 µL calcium chloride and kept in a shaking incubator (Miprolab) at 37°C for 2 min. To simulate the gastric digestion, 7.5 mL of gastric fluid, 1.6 mL of pepsin (25,000 U/mL), and 5 µL of calcium chloride were added to the mixture, and the pH of the mixture was adjusted to 3 using 1 M HCl. It was then incubated at 37°C for 2 h, and at the end of this period, 5 mL of sample was taken from the gastric phase. Then, for simulation of intestinal digestion, 8.25 mL of intestinal fluid, 3.75 mL of pancreatin (800 U/mL), 1.875 mL of bile (160 mM), and 30 µL calcium chloride were added to the remaining mixture, and the pH of the mixture was adjusted to 7 with 1 M NaOH. The mixtures were placed back in the shaking incubator and incubated at 37°C for another 2 h. At the end of intestinal digestion, all samples were collected and centrifuged at 4000 rpm for 10 min and stored at −20°C for further spectrophotometric and HPLC analyses. Bioaccessibility was calculated by dividing the amount of phenolics/antioxidants in digested samples (after intestine) by the amount of phenolics/antioxidants in undigested samples and multiplying by 100.

### 2.9 Spectrophotometric analyses

Spectrophotometric analyses were performed using an UV‐visible spectrophotometer (VWR, UV‐3100 PC). Total phenolic content (TPC) was determined according to the Folin–Ciocalteau method (Singleton et al., 1999) and values were expressed as mg gallic acid equivalents (GAE) per 100 g dm. Total flavonoid content (TFC) was measured by the method of Dewanto et al. (2002) and the results were expressed as mg catechin equivalents (CE) per 100 g dm. Total antioxidant activity (TAA) of gluten‐free muffins was estimated using 2,2‐diphenyl‐1‐picrylhydrazyl (DPPH) method previously described by Rai et al. (2006) and the CUPRAC (copper reducing antioxidant capacity) method as described by Apak et al. (2004). The results for two antioxidant methods were expressed in terms of mg Trolox equivalent (TE) per g dm. The analyses were performed in thrice.

### 2.10 Quantification of phenolic profile using HPLC

Phenolic profile of undigested and digested gluten‐free muffins was quantified using high‐performance liquid chromatography (HPLC, Waters 2695, W600 Waters), following the method of Bino et al. (2005). The phenolic compounds detection was performed at 280 nm for gallic acid, vanillic acid, protocatechuic acid, hydroxybenzoic acid, and benzoic acid derivatives, 312 nm for ferulic acid and chlorogenic acid, and 360 nm for quercetin derivatives. The concentrations of all identified phenolic compounds were expressed as µg per g dm.

### 2.11 Statistical analysis

The analytical experiments were conducted with three technical replicates. One‐way analysis of variance was performed with MINITAB software (MINITAB 18, Minitab Inc.) and the differences between samples were determined by Tukey test (*p *< 0.05). Correlations between TPC, TFC, and TAA analyses were evaluated using the Pearson correlation coefficient, calculated in Microsoft Excel for Mac Version 16.52 (MS Office).

## 3 RESULTS AND DISCUSSION

### 3.1 Physical properties of gluten‐free muffins

The physical properties (weight loss, height, volume index, symmetry index, and uniformity index) of gluten‐free muffins are shown in Table 2. The substitution of quinoa and rice flour with artichoke leaves caused a decrease in weight loss compared to control, however, the incorporation of GLP to the gluten‐free muffins did not significantly affect the weight loss (*p* > 0.05). This could be due to the high dietary fiber content of artichoke leaves, which delays the migration of water from the crumb to the crust during baking (Miranda‐Villa et al., 2019). Matos et al. (2014) also reported that rice flour‐based muffins enriched with different protein sources (soy protein, pea protein, egg white protein, casein, and wheat gluten) did not significantly affect the weight loss of the muffins. The height of the gluten‐free muffins did not change significantly among the samples (*p* > 0.05). The gluten‐free flours like quinoa and rice flour generally result in lower volume compared to those containing gluten, which contributes to muffin volume (Barakat et al., 2024). The increase was observed in volume index when GLP isolate added to the formulation. However, it was determined that the volume index values of muffins decreased significantly when 15% artichoke leaves added to the formulation compared to sample PC. This could be due to the high dietary fiber content of artichoke leaves (85 g 100 g^−1^ dm) (Boubaker, Omri, et al., 2016; Çelik & Isik, 2023). The symmetry index is a measure of surface contour, where high values indicate that the cakes are taller in the center than the edges, while low values suggest a reduction in cake volume toward the end of the baking process (Ceylan et al., 2021). Thus, the symmetry index can provide insight into the batter's gas‐holding capacity during the final stage of baking. The incorporation of GLP and artichoke leaves did not change symmetry index values of the gluten‐free muffins. Heo et al. (2019) also found that there is no difference in symmetry index for muffins enriched with kimchi by‐product powder. The uniformity index of gluten‐free muffins was affected adversely by the addition of 15% artichoke leaves compared to control. For optimal muffin quality, the uniformity index should be as close to zero as possible (Ceylan et al., 2021). Therefore, the best uniformity index value was observed in control sample, which was the closest result to zero. However, the uniformity index went away from zero when the substitution ratio of artichoke leaves increased compared to control.

**TABLE 2:** Physical properties of gluten‐free muffin samples.

Sample	Weight loss (%)	Height (mm)	Volume index (mm)	Symmetry index (mm)	Uniformity index (mm)
**C**	9.6 ± 0.8A	30.0 ± 1.6A	125.0 ± 4.2B	43.2 ± 4.2A	1.1 ± 1.1B
**PC**	8.9 ± 0.6AB	30.4 ± 2.6A	140.2 ± 9.5A	32.7 ± 6.4A	3.0 ± 1.5AB
**AL5**	8.1 ± 1.5B	32.5 ± 1.8A	138.0 ± 9.4A	40.4 ± 8.9A	2.5 ± 1.0AB
**AL10**	8.2 ± 0.2B	32.2 ± 1.4A	132.5 ± 6.3AB	41.5 ± 6.3A	2.7 ± 1.2AB
**AL15**	7.7 ± 0.4B	30.1 ± 1.7A	124.8 ± 4.2B	36.0 ± 7.3A	4.5 ± 1.0A

*Note*: Values are presented as means ± standard deviation. Means labeled with different uppercase letters (A > B > C) within the same column indicate significant differences among gluten‐free muffins (*p* < 0.05).

Abbreviations: AL5, muffin with 10% GLP and 5% artichoke leaves; AL10, muffin with 10% GLP and 10% artichoke leaves; AL15, muffin with 10% GLP and 15% artichoke leaves; C, control muffin without green lentil protein (GLP) and artichoke leaves; PC, muffin with 10% GLP.

**FIGURE 1:**
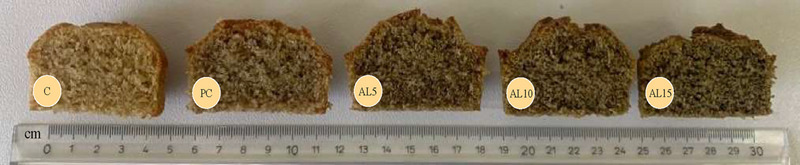
Vertical sections of the gluten‐free muffins containing different amounts of artichoke leaves. C: Control muffin without green lentil protein (GLP) and artichoke leaves; PC: muffin with 10% GLP; AL5: muffin with 10% GLP and 5% artichoke leaves; AL10: muffin with 10% GLP and 10% artichoke leaves; AL15: muffin with 10% GLP and 15% artichoke leaves.

Color and appearance are important parameters of the baked foods because it affects to the consumer's perception to the acceptability of the product. Figure 1 illustrates the cross‐sections of vertically cut muffins containing different amount of artichoke leaves. The increase in the addition of artichoke leaves ratio, resulting in a dense matrix and collapsed the structure due to the high fiber amount of artichoke leaves. These results also similar to those observed by Heo et al. (2019), who studied muffins with added kimchi by‐product.

The crust and crumb color values of gluten‐free muffins were given in Table 3. As the amount of artichoke leaves substitution ratio increased, the crumb and crust color changed significantly (*p* < 0.05). The crust color of muffins affected mainly by the Maillard and caramelization reactions (Ceylan et al., 2021), whereas the crumb color is mainly influenced by the artichoke leaves because the temperature of the muffin crumb does not rise to high temperature to accelerate the Maillard reaction (Heo et al., 2019). The L* and b* values of crumb and crust color revealed a significant decrease by adding GLP and artichoke leaves. The a* value of crumb color significantly increased when GLP added to the gluten‐free muffin formulation, whereas no significant change was observed in a* value with the increased substitution ratio of artichoke leaves (*p* > 0.05) compared to control sample. The a* value of crust color was not significantly changed by addition of GLP, while it significantly decreased when 15% of artichoke leaves added to the formulation (*p* < 0.05). Similar results were found by Boubaker, Damergi, et al. (2016) in parameters of crumb and crust color of bread enriched with artichoke by‐products. The ∆E* was calculated to assess any color differences perceptible to the human eye between the control and gluten‐free muffins enriched with GLP and artichoke leaves. Therefore, it can be concluded that the increased substitution ratio of artichoke leaves had significant effect on the color differences between control and other gluten‐free muffins.

**TABLE 3:** Crumb and crust color of gluten‐free muffin samples containing artichoke leaves.

	Crumb color				Crust color			
	L*	a*	b*	∆E*	L*	a*	b*	∆E*
**C**	56.5 ± 1.5A	1.7 ± 0.4B	31.8 ± 1.3A	–	51.3 ± 4.1A	9.6 ± 2.9A	38.8 ± 3.1A	–
**PC**	50.4 ± 1.3B	2.5 ± 0.3A	27.0 ± 0.5B	8.0 ± 1.8C	46.4 ± 3.1B	10.0 ± 2.7A	33.3 ± 2.0B	7.5 ± 1.0C
**AL5**	44.5 ± 1.4C	1.6 ± 0.5B	26.4 ± 0.7B	13.5 ± 1.5B	43.2 ± 3.2B	9.6 ± 2.2A	28.8 ± 2.3C	14.1 ± 3.0B
**AL10**	41.2 ± 0.8D	1.5 ± 0.5B	25.8 ± 1.0BC	16.7 ± 1.0A	41.0 ± 2.0CD	7.2 ± 1.8AB	25.8 ± 1.5C	17.2 ± 1.0AB
**AL15**	40.6 ± 1.2D	1.2 ± 0.4B	24.8 ± 0.7C	17.6 ± 0.9A	37.1 ± 2.3D	5.0 ± 1.3B	25.3 ± 0.9C	21.0 ± 3.2A

*Note*: Values are expressed as means ± standard deviation. Means with different uppercase letters (A > B > C) within the same column represent significant differences between gluten‐free muffins (*p* < 0.05).

Abbreviations: AL5, muffin with 10% GLP and 5% artichoke leaves; AL10, muffin with 10% GLP and 10% artichoke leaves; AL15, muffin with 10% GLP and 15% artichoke leaves; C, control muffin without green lentil protein (GLP) and artichoke leaves; PC, muffin with 10% GLP.

The textural parameters of gluten‐free muffins were investigated and results were represented in Table 4. The hardness values measured in samples containing artichoke leaves were significantly different from those in the control samples, C and PC (*p* < 0.05). An increase of hardness was observed for the analyzed gluten‐free muffin samples by addition of artichoke leaves, giving to the final product AL15 a total value of 22.7 ± 3.1 N due to the high dietary fiber of artichoke leaves. Dadalı (2023) reported that the hardness value of wheat cake containing 20% artichoke bracts as fat replacer found to be 10.51 N; however, the hardness value of wheat cake increased to the 53.94 N when 40% of artichoke bracts added as both fat and wheat replacers. Springiness measures elasticity and refers to the height that food regains during the interval between the end of the first compression and the beginning of the second compression (Shevkani & Singh, 2014). In this study, the addition of GLP and artichoke leaves to the muffins did not significantly change the springiness values among the samples (*p* > 0.05). Chewiness refers to the degree of difficulty involved in chewing food and forming a bolus before swallowing (Chiş et al., 2020). Statistical analysis revealed significant differences among the samples, indicating an improvement in chewiness with the addition of artichoke leaves and GLP (*p* < 0.05). This could be the ability of inulin to form gel and created a chewier texture (Laohaprasit & Sricharoenpong, 2018). Similar results were also obtained from a study conducted by Celik & Isik (2023). They reported significant increase in chewiness value of gluten‐free muffins with the addition of watermelon rind powder. Another study also demonstrated that the addition of wheatgrass and mung bean microgreens into the gluten‐free eggless rice muffins, the chewiness value increased (Kaur et al., 2022). Cohesiveness is closely related to the internal resistance of the food structure and the tendency of a sample to stick to itself. A high cohesiveness value is desirable to prevent the product from disintegrating during mastication (Chiş et al., 2020). The highest cohesiveness values were obtained from control sample and addition of GLP to the formulation decreased the cohesiveness values. However, the replacement of gluten‐free flour with artichoke leaves did not significantly change the cohesiveness values compared to control groups (*p* > 0.05). Gumminess is another textural parameter that is related to hardness and cohesiveness, and is determined by multiplying hardness by cohesiveness (Chiş et al., 2020). Gumminess values increased with the addition of artichoke leaves and showing the same patterns with hardness. Celik and Isik (2023) also reported an increase in the gumminess value of gluten‐free muffins as the watermelon rind substitution ratio increased.

**TABLE 4:** Textural profile of gluten‐free muffin samples.

Sample	Hardness (N)	Springiness (mm)	Chewiness (Nmm)	Cohesiveness	Gumminess (N)
**C**	11.5 ± 0.7D	8.7 ± 1.7A	34.8 ± 2.8D	0.3 ± 0.1A	4.1 ± 0.8C
**PC**	13.8 ± 1.6CD	9.5 ± 1.2A	39.5 ± 5.6CD	0.2 ± 0.0B	4.6 ± 1.0BC
**AL5**	16.4 ± 1.7BC	11.0 ± 2.0A	50.3 ± 7.3BC	0.2 ± 0.1AB	5.3 ± 1.0ABC
**AL10**	19.3 ± 2.4AB	9.9 ± 1.2A	65.5 ± 7.2A	0.2 ± 0.0AB	6.1 ± 0.7AB
**AL15**	22.7 ± 3.1A	9.1 ± 1.1A	57.0 ± 10.3AB	0.2 ± 0.0AB	6.6 ± 1.2A

*Note*: Values are presented as means ± standard deviation. Values with different uppercase letters (A > B > C) within the same column indicate significant differences between gluten‐free muffin samples (*p* < 0.05).

Abbreviations: AL5, muffin with 10% GLP and 5% artichoke leaves; AL10, muffin with 10% GLP and 10% artichoke leaves; AL15, muffin with 10% GLP and 15% artichoke leaves; C, control muffin without green lentil protein (GLP) and artichoke leaves; PC, muffin with 10% GLP.

### 3.2 Nutritional properties of gluten‐free muffins

Table 5 reported the nutritional properties of gluten‐free muffins. Moisture values ranged between 24.0% and 26.4%; however, there was no significant differences among samples. This could be due to the same baking parameters applied to the all samples. The lipid content of samples also did not show any significant differences by the addition of artichoke leaves. The lipid content of quinoa flour, rice flour, and artichoke leaves were determined as 5.0%, 1.4%, and 1.6%, respectively. So that, the main lipid source of muffins comes from the sunflower oil, which added the same amount into the all muffins. Ozgoren et al. (2019) also reported the similar results that addition of Jerusalem artichoke powder to the gluten‐free cracker did not significantly affect the lipid content. The protein content of sample C showed the lowest value compared to the other samples, and there were no significant differences between PC and other samples (AL5, AL10, and AL15) (*p* > 0.05). The protein content of green lentil was declared as 21.7% by manufacturer. After isolation, the protein content of GLP was determined as 89.3 ± 0.9% by Kjeldahl method. The increase in protein content of gluten‐free muffins must have been caused by the addition of GLP. This finding was compatible with the estimations; because GLP contains higher amount of protein content compared to quinoa and rice flour. Similar observations were also reported by Gularte, Gómez, et al. (2012) in gluten‐free cakes enriched with legume flours like chickpea, pea, lentil, and bean. The researchers determined that the protein content of the cakes increased with addition of legume flours by 1.4–1.5 compared to control sample. The ash content of gluten‐free muffins increased by 34% in the sample AL15 compared to control. On the other hand, carbohydrate content decreased significantly with the increasing supplementation of artichoke leaves. This reduction could be due to the replacement of quinoa and rice flour with artichoke leaves. Ozgoren et al. (2019) also reported the ash values of cracker samples increased and carbohydrate content decreased depending on the increasing amount of Jerusalem artichoke powder (0%–30%). Similar results were obtained by Ceylan et al. (2021) for cakes. The moisture and fat content did not change significantly; however, the ash and protein contents of cakes showed significant differences with the use of different ratios of Jerusalem artichoke flour.

**TABLE 5:** Nutritional properties of gluten‐free muffin samples.

Sample	Moisture (%)	Lipid (%)	Protein (%)	Carbohydrate (%)	Ash (%)
**C**	24.0 ± 0.4A	24.4 ± 0.8A	11.5 ± 0.6B	62.3 ± 0.3A	1.8 ± 0.1B
**PC**	26.1 ± 1.8A	23.6 ± 0.9A	15.8 ± 0.9A	58.5 ± 0.1B	1.9 ± 0.1B
**AL5**	24.5 ± 1.7A	24.8 ± 0.6A	17.0 ± 0.6A	56.5 ± 0.3BC	1.9 ± 0.0B
**AL10**	25.5 ± 0.7A	24.5 ± 3.0A	15.2 ± 0.9A	58.5 ± 0.6B	2.0 ± 0.1B
**AL15**	26.4 ± 0.9A	25.0 ± 0.9A	16.7 ± 1.0A	55.3 ± 1.1C	2.4 ± 0.0A

*Note*: Values are presented as means ± standard deviation. Means with different uppercase letters (A > B > C) within the same column represent significant differences between gluten‐free muffin samples (*p* < 0.05).

Abbreviations: AL5, muffin with 10% GLP and 5% artichoke leaves; AL10, muffin with 10% GLP and 10% artichoke leaves; AL15, muffin with 10% GLP and 15% artichoke leaves; C, control muffin without green lentil protein (GLP) and artichoke leaves; PC, muffin with 10% GLP.

### 3.3 The changes in TPC and TFC during in vitro gastrointestinal digestion

The effects of in vitro digestion on TPC and TFC of gluten‐free muffins were given in Table 6. For undigested samples, C and PC were found to contain lower amounts of TPC and TFC compared to undigested gluten‐free muffins enriched with artichoke leaves. The TPC and TFC levels gradually increased with the increasing proportion of artichoke leaves; however, there was no statistical difference between C, PC, and AL5. The highest TPC and TFC levels were found in gluten‐free muffins enriched with 15% artichoke leaves in the undigested sample. This result can be explained by the higher TPC of artichoke leaves, measured as 1762.6 ± 75.3 mg GAE 100 g^−1^ dm, compared to quinoa (274 mg ferulic acid 100 g^−1^) and rice (74.93–135.5 mg ferulic acid 100 g^−1^ dm) flours (de Mira et al., 2009; Hemalatha et al., 2016). After in vitro gastric digestion, TPC and TFC values did not exhibit a clear trend, with some samples showing enhancement and some demonstrating no change compared to undigested samples. After intestinal digestion, most samples exhibited higher TPC values compared to those observed undigested samples and the bioaccessibility of phenolic compounds was between 1.4‐fold and twofold enhanced after intestinal digestion compared to TPC in the undigested samples. After intestinal digestion, the TFC values sharply decreased in sample C, while the sample, AL5, showed enhancement and others demonstrated no significant change after in vitro intestinal digestion compared to gastric fraction. The enhancement in the TPC and TFC values can be attributed to the breakdown of linkages between phenolic compounds found in artichoke leaves and cell wall structures during intestinal digestion. Phenolic compounds can interact not only with dietary fiber but also with other macronutrients found in foods, such as carbohydrates, proteins, and lipids. Consequently, these compounds may be released from the food matrix in the upper gastrointestinal tract through the action of digestive enzymes and physiological fluids (Vaz et al., 2022). Additionally, the inclusion of artichoke leaves in gluten‐free muffins appears to increase the TPC and TFC in undigested samples, as indicated by data. These bioactive compounds are known for their antioxidant and potential health‐promoting properties. However, the bioactivity of these phenolics depends not only on their concentration in undigested samples but also on their stability, bioavailability, and activity during post‐digestion.

**TABLE 6:** Changes in the total phenolic content (TPC), total flavonoid content (TFC), and total antioxidant activity (TAA) of gluten‐free muffins during in vitro digestion.

Sample	Undigested	Gastric digestion	Intestinal digestion	Bioaccessibility
**TPC (mg GAE/100 g dm)**				
**C**	110.7 ± 3.5C,b	223.8 ± 9.2A,a	197.7 ± 16.4B,a	179%
**PC**	118.1 ± 9.7C,b	214.5 ± 13.2A,a	197.7 ± 12.4B,a	167%
**AL5**	132.3 ± 6.8C,b	179.0 ± 36.6A,b	272.8 ± 23.2AB,a	206%
**AL10**	181.7 ± 13.1B,b	254.9 ± 40.1A, ab	287.8 ± 51.2AB,a	158%
**AL15**	231.3 ± 29.1A,a	256.4 ± 66.1A,a	328.5 ± 0.9A,a	142%
**TFC (mg CE/100 g dm)**				
**C**	15.1 ± 1.8D,b	21.8 ± 3.3D,a	6.1 ± 0.4D,c	40%
**PC**	19.9 ± 1.0CD,a	25.2 ± 1.3D,a	25.2 ± 6.1C,a	127%
**AL5**	28.1 ± 3.1C,c	34.3 ± 2.5C,b	44.9 ± 2.4B,a	160%
**AL10**	61.5 ± 1.0B,a	57.1 ± 7.1B,a	70.4 ± 3.9A,a	115%
**AL15**	81.3 ± 10.7A,a	70.1 ± 0.9A,a	67.8 ± 6.2A,a	83%
**TAA**				
**CUPRAC (mg TE/100 g dm)**				
**C**	98.3 ± 9.6C,b	103.1 ± 16.3B,b	214.6 ± 8.8C,a	218%
**PC**	111.8 ± 3.7C,b	95.5 ± 2.9B,b	216.7 ± 12.9C,a	194%
**AL5**	156.5 ± 18.2C,b	84.6 ± 2.5B,c	343.9 ± 23.4B,a	220%
**AL10**	263.1 ± 22.7B,b	92.9 ± 8.0B,c	434.6 ± 22.1A,a	165%
**AL15**	346.2 ± 55.3A,b	161.4 ± 9.4A,c	492.7 ± 46.2A,a	142%
**DPPH (mg TE/100 g dm)**				
**C**	42.4 ± 5.2D,b	46.5 ± 6.1C,b	74.8 ± 9.7C,a	177%
**PC**	58.5 ± 5.4CD,b	54.8 ± 1.5C,b	95.6 ± 6.2C,a	164%
**AL5**	68.9 ± 4.9C,b	55.0 ± 6.0C,c	134.3 ± 7.2B,a	195%
**AL10**	101.2 ± 5.8B,b	91.9 ± 9.4B,b	193.0 ± 6.8A,a	191%
**AL15**	137.6 ± 18.8A,b	120.0 ± 13.5A,b	190.0 ± 28.0A,a	138%

*Note*: Values are presented as means ± standard deviation. Different uppercase letters (A > B > C) within the same column indicate significant differences between gluten‐free muffin samples, while different lowercase letters (a > b > c) within the same row indicate significant differences during in vitro digestion (*p* < 0.05).

Abbreviations: AL5, muffin with 10% GLP and 5% artichoke leaves; AL10, muffin with 10% GLP and 10% artichoke leaves; AL15, muffin with 10% GLP and 15% artichoke leaves; C, control muffin without green lentil protein (GLP) and artichoke leaves; CUPRAC, copper reducing antioxidant capacity; DPPH, 2,2‐diphenyl‐1‐picrylhydrazyl; PC, muffin with 10% GLP.

The bioaccessibility of phenolic compounds in muffins enriched with artichoke leaves appears to decrease with higher levels of artichoke leaves incorporation, as indicated by the results from in vitro digestion (Table 6). One possible explanation for the observed decrease in bioaccessibility with higher levels of artichoke leaves is the increased complexity of the muffin matrix. As the concentration of artichoke leaves rises, the muffins may become denser and more fibrous, which could hinder the release of phenolic compounds during digestion. This phenomenon has been noted in other studies where the incorporation of high‐fiber ingredients resulted in a more compact structure that may restrict the accessibility of bioactive compounds to digestive enzymes (Palafox‐Carlos et al., 2011). Moreover, the interactions between phenolic compounds and other components in the muffin, such as proteins and carbohydrates, may become more pronounced at higher levels of artichoke leaf inclusion. These interactions can form complexes that are less soluble and, therefore, less bioaccessible (Palafox‐Carlos et al., 2011).

### 3.4 Changes in TAA during in vitro gastrointestinal digestion

The differences in the TAA of gluten‐free muffins during in vitro gastrointestinal digestion are presented in Table 6. Among the undigested sample, AL15 was found to possess the highest antioxidant activity in both assays. For CUPRAC and DPPH assay results, gradual proportional substitution of quinoa and rice flour with artichoke leaves significantly increased TAA (*p* < 0.05); however, both assays showed that addition of GLP to formulations did not significantly change the TAA (*p* > 0.05). After gastric digestion, the outcomes varied, some showed reduction and others demonstrated no significant differences compared to undigested samples. The reduction in TAA after in vitro gastric digestion could be attributed to the specific modification in the phenolic compounds compared to undigested samples. pH variations can also lead to changes in antioxidant capacity, and the interaction of antioxidant phenolics with other components of the extracts may contribute to differences in bioaccessibility (Elejalde et al., 2024). In contrast, subsequent intestinal digestion resulted in an increased TAA. The results of both CUPRAC and DPPH assays generally indicated that the addition of artichoke leaves had a positive effect on the antioxidant activity of muffins. The increase in antioxidant activity observed during intestinal digestion may be due to the formation of new oxidant products that exhibit greater antioxidant activity than their precursors. Additionally, this enhancement could also be attributed to the presence of other antioxidant molecules that are not phenolic compounds (Kocakaplan et al., 2024).

Pearson correlation coefficients between TPC, TFC, and TAA were also determined (Table 7). Before in vitro gastrointestinal digestion, a highly linear relationship was obtained between TPC, TFC, and TAA, with the highest value being found between TFC and CUPRAC antioxidant assay (*R* ^2 ^= 0.998) followed by TPC and CUPRAC (*R* ^2 ^= 0.996). A highly correlation was also observed between TAA methods, CUPRAC and DPPH (*R* ^2 ^= 0.992). This positive linear relationship decreased after in vitro gastric digestion (*R* ^2 ^= 0.778), while continued after in vitro intestinal digestion (*R* ^2 ^= 0.973). The positive linear relationship that continued after intestinal digestion indicated that polyphenols contributed significantly to antioxidant capacity during digestion. Considering the pH conditions of the TAA methods applied in this study, it can be said that the CUPRAC and DPPH assays operating at physiological pH are more suitable for measuring antioxidant capacity after in vitro intestinal digestion. Therefore, it is recommended to apply more than one method with different mechanisms in measuring the TAA of foods (Kamiloglu, 2019).

**TABLE 7:** Pearson correlation coefficients between spectrophotometric methods.

Assays	Undigested	Gastric digestion	Intestinal digestion
TPC‐TFC	0.994	0.702	0.924
TPC‐CUPRAC	0.996	0.617	0.986
TPC‐DPPH	0.994	0.770	0.936
TFC‐CUPRAC	0.998	0.679	0.952
TFC‐DPPH	0.989	0.983	0.991
CUPRAC‐DPPH	0.992	0.778	0.973

Abbreviations: CUPRAC, copper reducing antioxidant capacity; DPPH, 2,2‐diphenyl‐1‐picrylhydrazyl; TPC, total phenolic content; TFC, total flavonoid content;

### 3.5 Changes in phenolic profile during in vitro gastrointestinal digestion

The effect of in vitro gastrointestinal digestion on the phenolic profile of the gluten‐free muffins can be seen in Table 8. The phenolic compounds with the highest concentration in undigested samples was *p*‐hydroxybenzoic acid, ferulic acid, and quercetin derivatives for C and PC samples, while gluten‐free muffins enriched with artichoke leaves, the concentrations of chlorogenic acid, quercetin derivatives, and benzoic acid derivative 1 significantly increased (*p* < 0.05). In the literature, the presence of quercetin and chlorogenic acid in artichoke wastes was reported by several authors (Ben Salem et al., 2017; Jiménez‐Moreno et al., 2019). Analyzing the concentration of individual phenolic compounds, the gastric fraction of each phenolic compound was similar to or higher than those quantified in the undigested gluten‐free muffins with the exception of ferulic acid, quercetin derivative 1 and 3, which were decreased after gastric digestion. However, some new phenolic compounds detected in the samples after gastric digestion, which were identified as gallic acid, benzoic acid derivative 2, vanillic acid, and ferulic acid derivative. This situation can be explained by the conversion or release mechanisms of phenolic compounds by phase I and phase II reactions, which affected by gastric enzymes and pH of the stomach (Gulsunoglu‐Konuskan & Kilic‐Akyilmaz, 2022; Wojtunik‐Kulesza et al., 2020). After intestinal digestion, variable results were obtained. For example, the amount of chlorogenic acid decreased by 23%–56% compared to the values obtained in the stomach for muffins enriched with artichoke leaves, while the amount of benzoic acid derivative 2 increased by up to 1.3–3.4 times. In the literature, the isomerization of chlorogenic acid to other caffeoylquinic acids during digestion has been reported (Tagliazucchi et al., 2012). The decreases observed in the amount of chlorogenic acid may also be related to the pH change and the presence of bile salts causing precipitation (Chethan & Malleshi, 2007). In addition, the instability of caffeoylquinic acids in aqueous solutions may have contributed to the decrease observed (Vallejo et al., 2004).

**TABLE 8:** Effect of in vitro digestion on phenolic profile of gluten‐free muffins enriched with artichoke leaves (µg g^−1^ dm).

		C	PC	AL5	AL10	AL15
	**Hydroxybenzoic acids**					
*p*‐Hydroxybenzoic acid	Undigested	149.6 ± 35.1A,a	125.1 ± 17.1B,a	112.4 ± 12.5B,a	103.4 ± 7.7A,a	118.6 ± 17.9A,a
	Gastric digestion	170.2 ± 26.2A, ab	197.9 ± 8.4A,a	168.2 ± 3.5A, ab	166.1 ± 0.1A, ab	124.5 ± 20.9A,a
	Intestinal digestion	77.2 ± 31.7A,a	61.1 ± 8.53C,a	103.8 ± 11.2B,a	137.8 ± 30.2A,a	110.6 ± 17.9A,a
	Bioaccessibility	52%	49%	92%	133%	93%
Benzoic acid derivative 1	Undigested	8.1 ± 0.5A,d	8.3 ± 0.1B,d	21.8 ± 4.1A,c	44.5 ± 3.9A,b	63.1 ± 1.7A,a
	Gastric digestion	24.6 ± 9.5A,a	21.7 ± 2.7A,a	28.2 ± 5.4A,a	40.4 ± 4.4A,a	67.6 ± 29.2A,a
	Intestinal digestion	9.2 ± 1.7A,b	16.5 ± 3.1AB,b	29.1 ± 2.1A, ab	48.4 ± 10.7A,a	46.3 ± 2.5A,a
	Bioaccessibility	114%	199%	134%	109%	73%
Benzoic acid derivative 2	Undigested	ND	ND	ND	ND	ND
	Gastric digestion	24.4 ± 0.0B,c	44.0 ± 1.0A, ab	37.2 ± 3.5B, bc	37.5 ± 5.6B, bc	57.9 ± 7.5A,a
	Intestinal digestion	65.1 ± 8.3A,b	70.3 ± 11.5A,b	112.4 ± 5.9A, ab	128.1 ± 19.2A,a	72.6 ± 13.7A,b
	Bioaccessibility	6510%	7030%	11,240%	12,810%	7260%
Gallic acid	Undigested	ND	ND	ND	ND	ND
	Gastric digestion	25.7 ± 1.2A, ab	30.1 ± 4.0A,a	24.1 ± 0.3A, ab	21.6 ± 2.1A,b	22.1 ± 2.0A, ab
	Intestinal digestion	8.3 ± 2.9B,a	6.4 ± 0.8B,a	9.7 ± 0.6B,a	11.3 ± 1.2B,a	12.4 ± 1.9B,a
	Bioaccessibility	830%	640%	970%	1130%	1240%
Protocatechuic acid	Undigested	15.2 ± 2.5B,a	15.5 ± 0.2C,a	18.1 ± 3.7B,a	14.4 ± 0.8B,a	22.2 ± 4.9C,a
	Gastric digestion	147.9 ± 18.0A,a	121.7 ± 0.6A,a	114.1 ± 7.5A,a	109.3 ± 15.9A,a	95.8 ± 22.4B,a
	Intestinal digestion	126.6 ± 1.7A, ab	80.3 ± 11.6B,b	140.9 ± 17.1A, ab	173.0 ± 35.1A,a	154.2 ± 3.0A, ab
	Bioaccessibility	833%	518%	778%	1201%	695%
Vanillic acid	Undigested	ND	ND	ND	ND	ND
	Gastric digestion	8.2 ± 0.9A,a	7.1 ± 2.3A,a	4.3 ± 0.8A,a	5.9 ± 4.1A,a	4.9 ± 0.6A,a
	Intestinal digestion	5.5 ± 0.5A, ab	3.4 ± 0.3A,b	7.3 ± 1.3A,a	6.6 ± 1.4A, ab	6.8 ± 0.3A, ab
	Bioaccessibility	550%	340%	730%	660%	680%
	**Hydroxycinnamic acids**					
Ferulic acid	Undigested	94.7 ± 6.2A,a	122.3 ± 14.4A,a	110.1 ± 6.9A,a	128.0 ± 28.5A,a	146.1 ± 5.4A,a
	Gastric digestion	71.7 ± 5.3B,a	80.4 ± 11.6A,a	70.4 ± 4.2B,a	60.2 ± 7.6AB,a	63.6 ± 8.6B,a
	Intestinal digestion	15.3 ± 1.2C,a	10.3 ± 0.2B,a	15.2 ± 0.7C,a	22.6 ± 9.5B,a	20.5 ± 0.5C,a
	Bioaccessibility	16%	8%	14%	18%	14%
Ferulic acid derivative	Undigested	ND	ND	ND	ND	ND
	Gastric digestion	14.7 ± 0.9A,a	14.9 ± 1.0A,a	18.1 ± 2.1A,a	18.4 ± 3.3A,a	22.3 ± 11.6A,a
	Intestinal digestion	13.7 ± 0.4B,c	2.4 ± 0.1B,c	5.3 ± 0.1B, bc	12.8 ± 4.7A, ab	15.7 ± 0.0A,a
	Bioaccessibility	1370%	240%	530%	1280%	1570%
Chlorogenic acid	Undigested	26.0 ± 0.9B,d	22.2 ± 0.9B,d	71.4 ± 3.7A,c	187.3 ± 5.9A,b	280.7 ± 23.1A,a
	Gastric digestion	37.0 ± 2.0A,c	35.9 ± 2.2A,c	79.8 ± 5.9A,c	170.6 ± 26.6A,b	295.3 ± 35.5A,a
	Intestinal digestion	28.6 ± 0.1B,d	24.8 ± 1.1B,d	52.8 ± 1.4B,c	99.2 ± 1.1B,b	129.9 ± 5.6B,a
	Bioaccessibility	110%	112%	74%	53%	46%
	**Flavonoids**					
Quercetin derivative 1	Undigested	68.2 ± 6.6A,b	75.1 ± 1.0A, ab	155.5 ± 47.5A, ab	205.3 ± 65.8A, ab	259.1 ± 66.0A,a
	Gastric digestion	33.6 ± 2.9B,b	43.3 ± 1.0B, ab	36.8 ± 4.8B,b	51.7 ± 8.1AB, ab	75.6 ± 19.1B,a
	Intestinal digestion	10.1 ± 0.1C,c	6.2 ± 0.6C,c	12.6 ± 2.7B, bc	37.4 ± 7.1B, ab	44.1 ± 12.1B,a
	Bioaccessibility	15%	8%	8%	18%	17%
Quercetin derivative 2	Undigested	93.1 ± 20.3A,b	98.2 ± 13.5A,b	103.3 ± 6.5A,b	223.9 ± 88.2A,b	443.1 ± 35.1A,a
	Gastric digestion	84.6 ± 1.3A,a	97.4 ± 11.0A,a	97.9 ± 11.3A,a	67.4 ± 3.4A,a	79.2 ± 19.6A,a
	Intestinal digestion	19.6 ± 2.1B,b	9.6 ± 1.6B,b	13.6 ± 0.2B,b	63.6 ± 16.8A,a	70.9 ± 3.9B,a
	Bioaccessibility	21%	10%	13%	28%	16%
Quercetin derivative 3	Undigested	43.5 ± 6.1A,c	48.6 ± 3.6A, bc	77.5 ± 14.1A, bc	141.3 ± 31.6A, ab	202.0 ± 41.1A,a
	Gastric digestion	21.3 ± 1.0B,a	20.9 ± 2.3B,a	17.8 ± 2.1B,a	22.5 ± 1.8B,a	19.7 ± 4.1B,a
	Intestinal digestion	12.2 ± 0.8B,b	11.9 ± 0.4B,b	14.8 ± 1.5B,b	26.5 ± 5.3B, ab	33.2 ± 8.1B,a
	Bioaccessibility	28%	25%	19%	19%	16%

*Note*: Values are presented as means ± standard deviation. Different uppercase letters (A > B > C) in the same column indicate significant differences within each phenolic compound across the undigested, post‐gastric and post‐intestinal digestion stages, while different lowercase letters (a > b > c) in the same row indicate significant differences between gluten‐free muffin samples (*p* < 0.05).

Abbreviations: AL5, muffin with 10% GLP and 5% artichoke leaves; AL10, muffin with 10% GLP and 10% artichoke leaves; AL15, muffin with 10% GLP and 15% artichoke leaves; C, control muffin without green lentil protein (GLP) and artichoke leaves; PC, muffin with 10% GLP.

The bioaccessibility of individual phenolic compounds in gluten‐free muffins enriched with artichoke leaves was assessed through in vitro digestion (Table 8), revealing significant insights into the stability and potential health benefits of these compounds during gastrointestinal processing. The results indicate that the bioaccessibility of various phenolic acids, including hydroxybenzoic and hydroxycinnamic acids, varies considerably across different stages of digestion phases. For instance, *p*‐hydroxybenzoic acid exhibited a notable decrease in concentration during intestinal digestion, with a bioaccessibility of 52% in the sample C, while increase in sample AL10 with a bioaccesibility of 133%. Benzoic acid derivative 2, gallic acid, vanillic acid and ferulic acid derivative although initially undetectable in the undigested muffins, demonstrated a significant bioaccessibility after intestinal digestion. This finding highlights the potential for certain phenolic compounds to be released and absorbed more effectively during digestion, which may enhance their health‐promoting effects. The hydroxycinnamic acids, particularly ferulic acid, displayed lower bioaccessibility rates, showing only 14%, 18%, and 14% bioaccessibility in the sample AL5, AL10, and AL15, respectively. Moreover, the presence of flavonoids, such as quercetin derivatives, also demonstrated varying degrees of bioaccessibility, with quercetin derivatives showing only 15%–28% in undigested sample and changing between 8% and 28% in muffins enriched with artichoke leaves. The varying levels of bioaccessibility among different phenolic compounds indicate that the structural characteristics and interactions within the muffin matrix play a crucial role in their release during digestion.

## 4 CONCLUSION

Gluten‐free products generally have a limited product range and are often deficient in beneficial nutrients. This study aimed to expand the variety of gluten‐free options available to individuals with celiac disease and to enhance their nutritional content by incorporating functional nutrients from artichoke leaves and GLP isolate. The incorporation of artichoke leaves and GLP significantly increased the ash and protein content of gluten‐free muffins. Furthermore, the results demonstrated that incorporating artichoke leaves enhanced the TPC, TFC, and TAA of the muffins. The addition of artichoke leaves was also improved the amount of chlorogenic acid and quercetin derivatives. Consequently, future research should focus on improving the sensory qualities, possibly through ingredient modifications or processing techniques, to better balance the health benefits with consumer acceptance.

## AUTHOR CONTRIBUTIONS

**Zehra Gulsunoglu‐Konuskan**: Conceptualization; investigation; funding acquisition; writing—original draft; methodology; writing—review and editing; formal analysis; supervision; data curation. **Sevgi Deren Yagdi**: Methodology; formal analysis; writing—original draft. **Burcu Ersoy**: Methodology; formal analysis.

## CONFLICT OF INTEREST STATEMENT

The authors declare no conflicts of interest.

## Data Availability

The authors confirm that the data will be made available on reasonable request.
